# Online Measurement System for Dynamic Flow Bioreactors to Study Barrier Integrity of hiPSC-Based Blood–Brain Barrier In Vitro Models

**DOI:** 10.3390/bioengineering9010039

**Published:** 2022-01-16

**Authors:** Jihyoung Choi, Sanjana Mathew, Sabrina Oerter, Antje Appelt-Menzel, Jan Hansmann, Tobias Schmitz

**Affiliations:** 1 Department of Tissue Engineering and Regenerative Medicine, University Hospital Würzburg, Röntgenring 11, 97070 Würzburg, Germany; sanjana.mathew@uni-wuerzburg.de (S.M.); Jan.Hansmann@uni-wuerzburg.de (J.H.); tobias.schmitz@uni-wuerzburg.de (T.S.); 2 Translational Center for Regenerative Therapies, Fraunhofer Institute for Silicate Research, Röntgenring 11, 97070 Würzburg, Germany; sabrina.oerter@isc.fraunhofer.de; 3 Faculty of Electronics, University of Applied Science Würzburg-Schweinfurt, Ignaz-Schön-Straße 11, 97421 Schweinfurt, Germany

**Keywords:** dynamic flow bioreactor, electrochemical impedance spectroscopy, transepithelial/transendothelial electrical resistance, blood–brain barrier (BBB), human induced pluripotent stem cells (hiPSCs), brain capillary-like endothelial cells (BCECs), shear stress

## Abstract

Electrochemical impedance spectroscopy (EIS) is a noninvasive, reliable, and efficient method to analyze the barrier integrity of in vitro tissue models. This well-established tool is used most widely to quantify the transendothelial/epithelial resistance (TEER) of Transwell-based models cultured under static conditions. However, dynamic culture in bioreactors can achieve advanced cell culture conditions that mimic a more tissue-specific environment and stimulation. This requires the development of culture systems that also allow for the assessment of barrier integrity under dynamic conditions. Here, we present a bioreactor system that is capable of the automated, continuous, and non-invasive online monitoring of cellular barrier integrity during dynamic culture. Polydimethylsiloxane (PDMS) casting and 3D printing were used for the fabrication of the bioreactors. Additionally, attachable electrodes based on titanium nitride (TiN)-coated steel tubes were developed to perform EIS measurements. In order to test the monitored bioreactor system, blood–brain barrier (BBB) in vitro models derived from human-induced pluripotent stem cells (hiPSC) were cultured for up to 7 days. We applied equivalent electrical circuit fitting to quantify the electrical parameters of the cell layer and observed that TEER gradually decreased over time from 2513 Ω·cm^2^ to 285 Ω·cm^2^, as also specified in the static control culture. Our versatile system offers the possibility to be used for various dynamic tissue cultures that require a non-invasive monitoring system for barrier integrity.

## 1. Introduction

One of the main characteristics of endothelial and epithelial cells is their ability to form polarized and tight cellular barriers due to the presence of various tight junctions (TJs), adherence junctions, and junction adhesion proteins at intercellular clefts [1].

This barrier integrity is required to maintain tissue homeostasis and to regulate various transport mechanisms via transporter proteins [2]. Disruption of barrier integrity often leads to tissue-specific dysfunction, which is implicated in several human diseases [3]. An intact barrier is a crucial requirement for in vitro studies based on engineered barrier tissues such as skin, the gastrointestinal tract, or the endothelium of the blood–brain barrier (BBB) [4,5,6]. The barrier integrity is often determined via permeability tests using various molecular weight tracers or a trans-endothelial/-epithelial electrical resistance (TEER) measurement [7,8].

TEER is an electrical parameter representing electrical resistance across the tight cellular layers of barrier tissues [9,10]. Barrier integrity and quality of in vitro models can be efficiently quantified by non-invasive measurement of TEER-based techniques without interfering with cellular functionality [9,11,12]. Therefore, TEER values are determined by resistance meters and electrochemical impedance spectroscopy (EIS) [13,14,15,16].

In static cellular in vitro models, TEER is mostly measured using commercially available resistance meters and handheld chopstick electrodes such as EVOM and Millicell ESR-2. Although resistance-based TEER measurement is a valuable non-invasive technique for the measurement of barrier integrity, allowing for fast and easy read-outs, it is prone to errors and variations as it is affected by parameters such as temperature, cell culture medium, cell passage numbers, cell culture period, and shear stress [8]. EIS on the other hand is an advanced technique for measuring impedance by applying frequency-dependent alternating currents. In contrast to read-outs of resistance meters based on single frequency measurements, a frequency spectrum over a defined range is recorded. Regarding EIS in a cell culture setup, typically recordings over a frequency spectrum from 1 to 100 kHz are performed, offering the possibility to obtain information not only about the cellular system (amplitude of the resistance and capacitance), but also about the measuring electrodes and cell culture medium [9,13]. Simulation software, and fitting an electric equivalent circuit to the recorded cellular impedance, supports the most accurate values for each electric element in the measuring setup and the cellular model. EIS is therefore a well-established tool to analyze the barrier integrity of Transwell-based models under static culture conditions.

However, in comparison to static cell culture, the application of dynamic culture in bioreactors facilitates advanced cell culture conditions that mimic a more tissue-specific environment additionally providing physical cues and improved nutrient supply [17]. The continuous non-invasive monitoring of cell culture models cultivated under dynamic conditions is as important as under static conditions. Some studies have already shown that EIS is a suitable technique for application under dynamic culture conditions [18,19,20]. In these studies, EIS was applied to monitor cell layers in microfluidic devices using electrodes that were directly integrated during the manufacturing process of these devices. However, it is still necessary to consider how to implement non-invasive EIS monitoring in different bioreactor systems for dynamic cell culture [21], especially if the bioreactor’s manufacturing process should not be further complicated or the possibility to measure non-invasively should be later added to already existing devices.

One example for a tissue model that could benefit from both dynamic culture in a bioreactor as well as monitoring by EIS is an in vitro model of brain capillary-like endothelial cells (BCECs).

BCECs differ from peripheral endothelial cells due to their low pinocytotic activity, increased mitochondrial activity, absence of fenestrae, complex TJ network, and specialized transport mechanisms that allow a highly regulated movement of substances to the brain and vice versa, thereby regulating cerebral homeostasis and prevention of toxin and pathogen entry into the brain [22,23,24,25]. These special barrier characteristics of BCECs additionally act as a hurdle in pharmaceutical drug development aiming at increased bioavailability of pharmaceuticals to the brain. To meet the requirements of the pharma industry as well as to circumvent species-related differences in pharmaceutical testing, human in vitro BBB models are being developed [4]. We previously established a static Transwell-based in vitro model of the BBB using hiPSC-derived cell types [26]. This in vitro model is characterized by physiological BBB characteristics. However, the demands of shear stress-sensitive BCECs are not met in static culture conditions as in vivo they are subjected to a fluidic shear stress with a rate of ~5 to 23 dyne/cm^2^ [27,28].

Several reported dynamic flow bioreactor systems are promising for the provision of physiologically relevant in vitro culture conditions in advanced BBB modelling [15,29,30]. The current focus of dynamic hiPSC-derived BBB models is on the development of organ-on-a-chip platforms, and most of them do not offer the possibility to measure TEER online [31,32,33]. The main challenges faced here are the reproducibility of culture conditions, cost efficiency in production, easy handling in bioreactor setup, and integration of non-invasive measurement systems for cellular readouts during culture periods [34].

The aim of our study was, firstly, to produce a cost-efficient dynamic flow bioreactor having attachable electrodes allowing for constant non-invasive monitoring of cellular monolayers cultured under dynamic conditions. In a previous study, we could show that nano-rough titanium nitride (TiN) electrodes enhanced impedance measurement sensitivity; therefore we aimed to also apply this material under dynamic measurement conditions [35]. We fabricated our system, comprised of bioreactor and electrodes, using computer-aided design (CAD) modelling, 3D printing, polydimethylsiloxane (PDMS) casting, oxygen plasma treatment, and physical vapor deposition (PVD). As a model example, we adapted a 7 days (168 h) culture of hiPSC-derived BCECs within the system and monitored barrier integrity online. With the aid of the novel dynamic flow bioreactor system and integrated EIS measurement developed in this study, we were able to efficiently and non-invasively monitor cellular barrier integrity. The BCECs, used as an in vitro model for BBB test systems, could be cultivated successfully for a period of 7 days under dynamic conditions, and cellular changes in comparison to standard static cultures could be identified.

## 2. Materials and Methods

### 2.1. Bioreactor Design and Fabrication

#### 2.1.1. Bioreactor Chambers from PDMS

**Figure 1:**
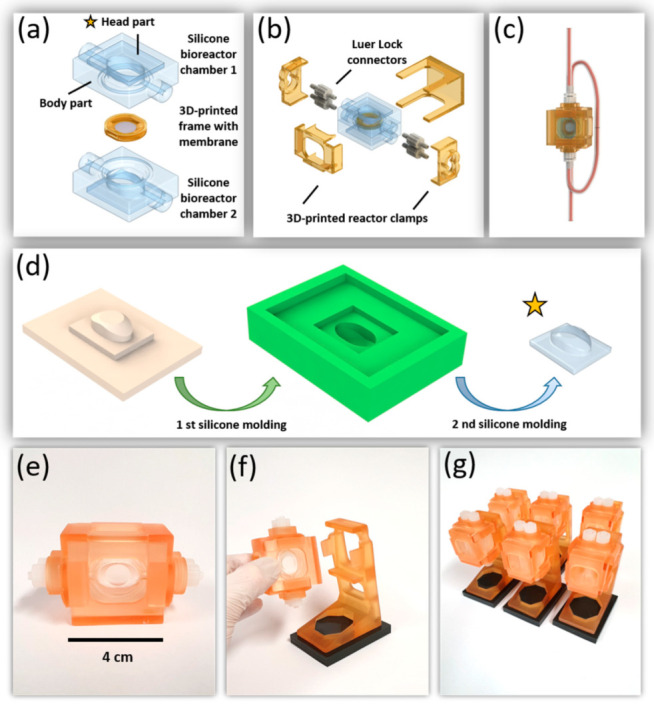
Design, fabrication process, and final realization of the chip bioreactor. (**a**) CAD image of the two bioreactor chambers, each comprising a bonded head and body part, together with the 3D-printed frame; (**b**) explosion view of the complete reactor with four 3D-printed clamps and Luer Lock connectors; (**c**) CAD image of the assembled bioreactor with attached tubing; (**d**) the silicone parts of the bioreactor are fabricated by a combination of 3D printing and two silicone molding steps. Head and body parts were then bonded by stick and stamp method (marked by yellow star) to create a bioreactor chamber; (**e,f**) photographs of the final bioreactor with and without holder; (**g**) parallelization of all the involved fabrication processes allowed fast small batch productions of the silicone bioreactors and the necessary equipment.

For a cost-efficient, reproducible, and highly available bioreactor, two bioreactor chambers were made out of polydimethylsiloxane (PDMS) (Figure 1a). Each bioreactor chamber was composed of body and head parts. The parts were produced by following three fabrication steps. In the first fabrication step of the PDMS bioreactors, the different parts of the reactors were designed using SOLIDWORKS™ 3D design software (Dassault Systems, Stuttgart, Germany). The designed parts were then 3D printed by an SLA 3D printer (Dental Model resin FLDMB01, Form 2; Formlabs GmbH, Berlin, Germany). SLA-printed parts were washed in isopropanol (Carl Roth GmbH, Kalsruhe, Germany) for 10 min using the Form Wash device (Formlabs GmbH, Berlin, Germany). Afterwards, the parts were cured with UV light at 60 °C for 30 min using the Form Cure device (Formlabs GmbH, Berlin, Germany). Before being used for silicone molding, the parts underwent print post-processing by fine-grained sandpaper (SiC sandpaper #2400; Schmitz Metallographie, Germany). In the second step, the silicone molds were produced by molding the 3D-printed parts with a special molding silicone (DUBLISIL^®^15; Dentalversender, Cologne, Germany). After hardening of the molded silicone (30 min, room temperature) and removal of the 3D-printed parts, the molds underwent a 2 min oxygen plasma treatment (500 W, 0.3 mbar, 12 sccm) in a plasma chamber (Pico Plasma System; Diener Electronics, Ebhausen, Germany). In the third step, PDMS polymer (Sylgard^®^ 184; Dow Corning, Wiesbaden, Germany) was mixed in a ratio of 10:1 (pre-polymer and cross linker) and air bubbles produced by mixing were removed using a vacuum chamber. PDMS was filled into 50 mL syringes and injected in the plasma-treated silicone molds with an injection rate of 1 mL/min using syringe pumps (World Precision Instrument, Friedberg, Germany). In order to accomplish PDMS polymerization, the silicone molds filled with the injected PDMS mixture were placed in an oven at 37 °C for 10 h. Finally, the PDMS parts were removed from their molds, and the body and head parts that form the bioreactor chambers were bonded together by the stick and stamp method. Hereby, a thin layer of fresh PDMS was applied, and the two parts were pressed together and finally treated in an oven at 100 °C for 2 h.

#### 2.1.2. Membrane Frame and Additional Equipment

Frames for holding and stabilizing the cell culture membranes were designed using SOLIDWORKS™ 3D design software (Dassault Systems, Stuttgart, Germany) and printed by a 3D printer (Form 2; Formlabs) using a biocompatible resin (Dental SG FLDGOR01; Formlabs, Berlin, Germany). The 3D-printed membrane frames were post-processed with cleaning (washing times 5 min), hardening, and a final surface refinement described in previous Section 2.2.1. Subsequently, the membrane frames were treated at least twice by ultra-sonification for 15 min in pure water. At last, parts were incubated for 15 min in 70% EtOH (Carl Roth GmbH, Kalsruhe, Germany) to remove any material residue from the printing process and then autoclaved (121 °C, 30 min). The membrane frame was composed of two fitting parts, allowing to sandwich the cell culture membrane (Oxyphen, Unique Mem Track Etched Membrane, 0.4 µm pore size, LOT (210401U4), Prototype PR0500-1708) without any creasing. To prepare a membrane chip for cell culture, the porous membrane was positioned in between the membrane frames and glued using a thin layer of PDMS. During gluing, the sandwiched porous membrane was stretched evenly by gently pressing the membrane frames, thereby creating a cell culture area of 0.95 cm^2^ (Figure 1a). As shown in Figure 1b,f, reactor clamps that tightened the bioreactors, and the bioreactor holder, were prepared by 3D printing with the biocompatible resin. In addition, the platforms for a secure positioning of the reactors were cast from molding silicone.

#### 2.1.3. COMSOL Multiphysics

**Figure 2:**
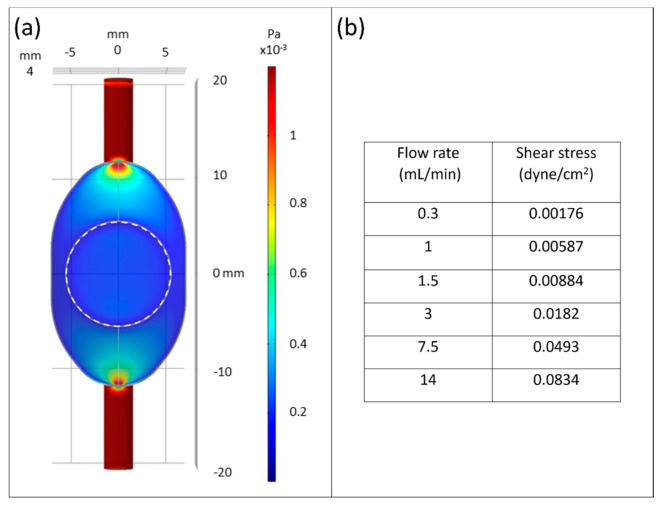
Fluidic computational simulation. (**a**) COMSOL simulation showed generation of homogeneous shear stress through the bioreactor with ~1.76 × 10^−3^ dyne/cm^2^ corresponding to 0.3 mL/min of medium flow rate over the cell culture area (dotted circular area); (**b**) flow rates in correlation to shear stress under stable laminar flow conditions.

COMSOL Multiphysics (Comsol Multiphysics GmbH, Berlin, Germany) was carried out to characterize fluid dynamic. Fluid domain was set as water with fluid density 1005.5 kg/m^3^ and kinematic viscosity 7.65 × 10^−3^ Pa·s. No-slip conditions were applied to boundary layer. For fluid dynamic simulation, flow conditions of 5.0 × 10^−9^ m^3^/s to 2.3 × 10^−7^ m^3^/s were set in inlet region and simulated (Figure 2).

### 2.2. Electrode Preparation

#### 2.2.1. Substrate Preparation and TiN Coating

**Figure 3:**
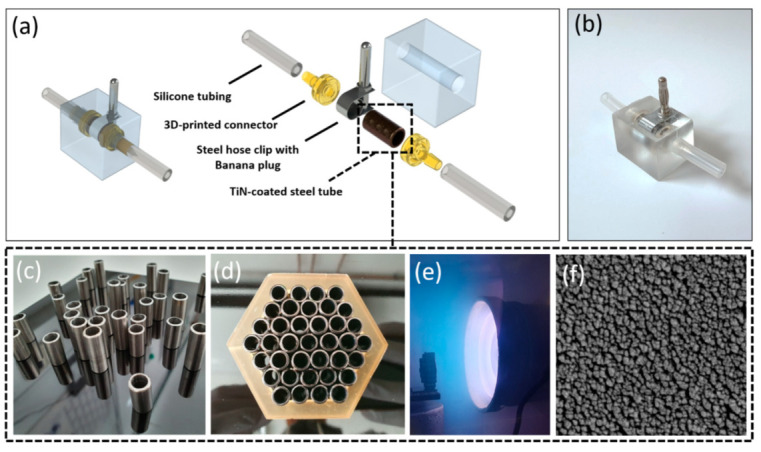
Design and fabrication process of TiN tube electrode. (**a**) Explosion view of the tube electrode system; (**b**) photograph of the fabricated tube electrode system; (**c**–**e**) steel tubes were cut, polished, and cleaned, before they were tightly packed, placed in the vacuum chamber, and finally coated by PVD with a TiN layer; (**f**) SEM image of the coating’s nano-rough TiN surface structure inside the tube electrodes.

Stainless steel tubes (Art. 4249598; Gust. Alberts GmbH & Co. KG, Herscheid, Germany) with an outer diameter (OD) of 8 mm and inner diameter (ID) of 6 mm were used as substrates for the electrodes. The stainless steel tubes were cut into a final length of 15 mm and the cut surface was polished (Figure 3c). Afterwards, the parts were washed in isopropanol and placed in the 3D-printed hexagonal frame to be fixed tightly (Figure 3d). TiN coating was deposited on the stainless steel substrates by physical vapor deposition (PVD) technique, as previously described [35]. Briefly, the sputtering target was made from titanium (12 cm diameter, 10 mm height), while argon (Ar) and nitrogen (N_2_) were used as a processing gas mixture with the pressure of the process gas set to 4.0 × 10^−3^ mbar during deposition. First, magnetron sputtering was carried out with a power of 800 W, which was generated by a 13.56 MHz RF generator (RF 1000; Hüttinger GmbH, Nuernberg, Germany) for 3 min. This was directly followed by a second sputtering process with a lower power of 500 W for 60 min (Figure 3e).

#### 2.2.2. Final Electrode Model

In order to install TiN-coated tube electrodes into the fluid flow circuit and to avoid any liquid leaks during medium circulation, suitable connectors and a PDMS shell for the electrodes were designed by CAD (Figure 3a). The connectors and a replica of the shell were designed, 3D printed, and processed as described in the first fabrication step of Section 2.1.1, with the biocompatible ‘Dental SG resin’ used for printing the connectors and ‘Dental Model resin’ for the model of the shell, respectively. Subsequently, the replica of the shell was used to perform silicone molding, as described in second fabrication step of Section 2.1.1. The 3D-printed connectors were connected to the TiN-coated steel tubes, and in addition, silicone tubes (30 mm length, ID 3.2 mm, OD 6.4 mm; Saint-Gobain, France) were attached to each connector. Furthermore, the outer surface of the TiN-coated tube electrodes was enclosed by a steel slotted hose clip (steel slotted hex bolt drive, Ø 7-9 mm; RS components GmbH, Frankfurt, Germany) with a banana plug (Ø 4 mm; Conrad, Hirschau, Germany) for the electrical connection (Figure 3a). These assembled parts were placed in a plasma-treated silicone mold (DUBLISIL^®^15; Dentalversender, Koeln, Germany) prepared previously, which was then filled with PDMS (Sylgard^®^ 184; Dow Corning, Wiesbaden, Germany). The PDMS was heat-cured at 55 °C for 3 h, afterwards, the completely assembled electrode was removed from its mold and was heated a second time at 80 °C for 2 h.

### 2.3. Electrode Characterization

#### 2.3.1. Electrical Characterization

The electrical properties of the TiN-coated tube electrodes were determined using EIS. For this characterization, two electrodes were connected with silicone tubes and filled with phosphate-buffered saline without calcium and magnesium (PBS^−^) electrolyte. The electrical characterization included testing of influencing factors, such as differing distances between the electrodes, as well as varying temperatures and flow rates. In detail, distances of 30, 60, 100, 150, and 200 mm between the electrodes were tested, as well as two temperatures (at fixed electrode distance of 60 mm) of 20 °C (room temperature) and 37 °C (optimal culture of human cells). Finally, in order to test the influence of the applied flow rate in the bioreactor system on the electrical measurement, two electrodes at a distance of 60 mm were connected to a peristaltic pump (Ismatec ISM 400 MS/CA 4-12; Cole-Parmer GmbH, Wertheim, Germany) and the temperature was constantly kept at 37 °C. Four different flow rates, in particular 0, 7.5, 140, and 280 mL/min, were used to investigate the impact of the flow rate on impedance. Impedance spectroscopy was carried out with an impedance spectrometer (PGSTAT204; Metro-ohm Autolab, Utrecht, The Netherlands). Alternating current (AC) was applied with 0.2 V_RMS_ in a frequency range of 1 Hz to 100 kHz.

#### 2.3.2. Scanning Electron Microscopy (SEM) Analysis

In order to observe the topography of the TiN coating inside of the stainless steel tubes they were first cut to quarters. The pieces were washed with acetone (Carl Roth, Karlsruhe, Germany) and finally coated with a 2 nm layer of platinum in a sputter coater (EM ACE600; Leica, Vienna, Austria). The image of the TiN coating inside of the stainless steel tube was then taken on a scanning electrode microscope (SEM; CB 340; Zeiss, Oberkochen, Germany), (Figure 3f).

### 2.4. Flow Bioreactor System

#### 2.4.1. Sterilization

**Figure 4:**
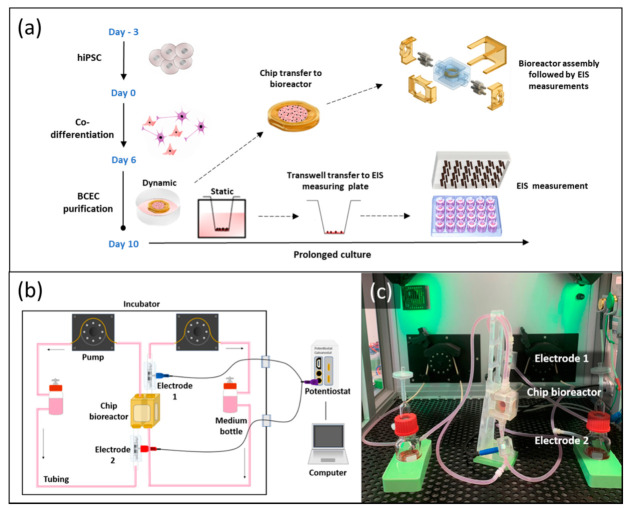
BCECs generation and EIS monitoring set up under static and dynamic conditions. (**a**) Overview of the differentiation process (from day 3 to day 10) of hiPSCs towards BCECs. On day 10 of differentiation, the membrane chip was transferred to a bioreactor system for dynamic culture; (**b**) schematic of bioreactor system; (**c**) photographical overview of the whole bioreactor system with attached electrodes for online monitoring of the dynamic culture of BCEC in vitro models.

The complete flow bioreactor system was composed of two medium bottles (capacity 50 mL), silicone tubes (OD 6.4 mm, ID 3.2 mm; Saint-Gobain, France), pump tubes (ID 2.79 mm; IDEX Health & Science GmbH, Wertheim, Germany), Luer Lock connectors, two TiN-coated tube electrodes, and the PDMS bioreactor. All the components were sterilized by autoclaving (121 °C, 30 min) prior to assembly (Figure 4c). The frame with the embedded cell culture membrane was sterilized by gamma sterilization prior to coating and seeding of the cells.

#### 2.4.2. Assembly of the Flow Bioreactor System

The sterilized components were connected in a biological safety cabinet. Assembling was performed as visualized in Figure 4c. The membrane chip pre-cultured with hiPSC-derived BCECs was inserted in between the bioreactor chambers and tightened with 3D-printed reactor clamps. Each chamber of the chip bioreactor was connected to individual flow regimes with a connected TiN-coated tube electrode. Each tube electrode was placed on the opposite side with the reactor in the middle. All silicone tubes were clamped tightly using screw clamps to prevent uncontrolled flow before starting the medium flow. Media bottles were filled with 45 mL of EC+ medium, each, and the whole system was moved to an in-house-adapted incubator system for dynamic culture and EIS measurements [36]. After installation, the screw clamps were removed and the peristaltic pump in the tailored incubator system was started with a flow rate of 0.3 mL/min. During the whole experiment, culture conditions in the incubator were kept constant at 37 °C and 5% CO_2_. Due to a large supply of medium (in total 90 mL pre bioreactor), medium was not exchanged over the culture period of 168 h in the bioreactor systems. Along with dynamic culture, the experiment on static culture in Transwells was carried out over the same period. EC medium change was performed daily.

### 2.5. Generation of hiPSC-Derived BCECs

The differentiation of hiPSC line IMR90-4 to BCECs was performed as described previously [26]. Briefly, hiPSCs were dissociated into single cells using Accutase™ (Sigma-Aldrich #A6964, Saint Louis, MO, USA) and seeded at an initial density of 7.5 × 10^3^ cells/cm^2^ in 10 cm^2^ dishes, coated with Matrigel (Corning #356231, Bedford, MA, USA) and treated for 24 h with mTeSR™1 medium (Stem Cell Technologies #85850) including 10 µM Y-27632 dihydrochloride (Toris #129830-38-2, Bristol, UK). Daily medium changes were performed with mTeSR™1 medium. When hiPSCs reached a density of 2.5–3.5 × 10^4^ cells/cm^2^, the medium was replaced by unconditioned medium with daily medium changes until day 6. On day 6 of differentiation, cells were treated with 4 mL/dish EC++ medium (human endothelial serum-free medium containing 1:200 B27 (Thermo Fischer Scientific #17504044, Bleiswijk, The Netherlands), supplemented with 10 μM all-trans retinoic acid (RA; Sigma-Aldrich, #R2625) and 20 ng/mL human basic fibroblast growth factor (hbFGF; PeproTech #100-18B, Cranbury, NJ, USA)) for two days. No medium change was performed on day 7.

On day 8 of differentiation, each membrane chip was firstly placed into one well of a 10 cm^2^ cell culture dish. Matrigel coating was performed freshly at a concentration of 200 µg/mL. Each Transwell (Greiner Bio-One #662641, Frickenhausen, Germany) was coated with 100 µL and each membrane chip was coated with 300 µL of Matrigel and incubated for 1 h at room temperature before cell seeding. On day 8, cells were detached with Accutase™ and seeded in EC++ medium at a density of 1 × 10^6^ cells/cm^2^ onto the pre-coated culture surfaces. Total apical medium amount used for cell seeding was 300 µL for the membrane chips and milk 200 µL for the Transwell inserts. Post-15 min adhesion time after cell seeding, 2 mL/10 cm^2^ of EC++ medium was gently added around each membrane chip. On day 9, medium was changed to EC medium (+B27, -RA, -hbFGF) for the rest of culture duration. For the Transwells, 400 µL of EC medium was used in the apical compartment and 850 µL was applied in the basolateral compartment, with daily medium changes. The membrane chips were gently inverted (cell upside down) into a 10 cm^2^ cell culture dish containing 3 mL of EC medium such that the cells were in direct contact with medium. Additionally, 300 µL of medium was gently added on top of each membrane, thereby ensuring no drying. On day 10 of differentiation, membrane chips were shifted to bioreactors.

### 2.6. EIS Analysis of In Vitro Models under Static and Dynamic Conditions

The impedance spectroscopy of static Transwell-based BCECs was carried out using a 2D measuring device with nanostructured TiN electrodes previously described by Schmitz et al. [35]. For EIS measurement, EC medium was filled in the apical (400 µL) and basolateral compartment (850 µL) of the culture plate containing Transwells, and incubated for 40 min at 37 °C to adapt the cells to the culture system and to ensure that effecting parameters are consistent between the measuring days. The impedance spectrometer PGSTAT204 (Metro-ohm Autolab, Utrecht, Netherlands) and the software program NOVA 2.1.3 (Metro-ohm Autolab, Utrecht, The Netherlands) were used for EIS measurement. The frequency range for measurements was 1 Hz to 100 kHz and a sinusoidal alternating current (AC) with an amplitude of 0.05 V_RMS_ was applied. NOVA software (Offenburg, Germany) was also used for the impedance data analysis and equivalent circuit fitting and simulation. The same set of parameters was applied for the EIS measurement of the dynamic system.

### 2.7. Immunofluorescence and Microscopy

Cells cultured on Transwell inserts and bioreactor membrane chips were washed once with PBS^-^ (Sigma-Aldrich, #D8537, Taufkirchen, Germany), fixed with 4% Roti Histofix (Carl Roth, # P087.2, Karlsruhe, Germany) for 15 min, and either covered with 1 mL of PBS^-^ and stored at 4 °C or used immediately for staining. The cells were permeabilized with 0.2% Trion X-100 (Carl Roth, #3051.2, Karlsruhe, Germany) in PBS^-^ for 5 min. The membranes were washed with washing buffer (0.5% Tween-20 prepared in PBS^-^, VWR # 9005-64-5, Darmstadt, Germany) followed by blocking for 20 min in blocking buffer (5% donkey serum, Biozol Diagnostica SBA-0030-01, Eching, Germany + 0.02% Saponin, Carl Roth # 8047-15-2, Karlsruhe, Germany + 0.1% TritonX-100 in PBS^-^). The primary antibodies ZO-1 (1:100; Proteintech, #21773-1-AP, Manchester, UK), GLUT-1 (1:200; Abcam #ab40084, Cambridge, UK), and Occludin (1:200; Thermo Fisher Scientific #33-1500, Bleiswijk, The Netherlands) were diluted in blocking buffer and samples were incubated overnight at 4 °C. The membranes were washed and incubated with secondary antibodies anti-rabbit Alexa Fluor 647 (Invitrogen #A-31573, Rockford, IL, USA) or anti-mouse Alexa Fluor 488 (Invitrogen #A-21206, Rockford, IL, USA) at RT for 1 h. The membranes were further washed and mounted with DAPI Fluoromount-G solution (Biozol Diagnostica, #SBA-0100-20, Eching, Germany). Maximum projection Z-stack images were acquired using a Leica SP8 confocal laser scanning microscope (Leica Microsystems, Wetzlar, Germany). In order to assess cell nuclei numbers, 40× maximum projection images were analyzed per condition (static 0 h, dynamic 168 h, and static 168 h). Nuclei from a total of 27 images (N = 3 biological replicates, N = 3 different areas per condition) were counted automatically using particle analyzer in Fiji [37].

## 3. Results

### 3.1. Flow Bioreactor System

The aim of this study was to develop a cost-efficient, reproducible, and simple assembly bioreactor system for dynamic conditions. In addition, this system should allow a simple transfer from static cell culture protocols to dynamic culture. Therefore, the developed bioreactor is based on the implementation of a membrane-holding frame (Figure 1a) with cell culture membrane. This concept supports in vitro culture of tissue models in well plates and monitoring of cellular layers prior to their shift to dynamic culture conditions. Subsequent transfer into the bioreactor system can be easily performed, since the membrane itself did not have to be directly handled, avoiding the risk of damaging confluent cellular layers. In static culture, the membrane frame was originally designed to be used as a flattened Transwell that could evenly stretch biological matrices and allows for pre-culturing of in vitro tissue models. Inserted in the bioreactor system, the membrane frame perfectly aligned with the surrounding PDMS chamber to create a homogenous medium flow over the culture surface, allowing optimal nutrient supply (Figure 2a).

The PDMS chip bioreactor design that was composed of three major parts, two PDMS bioreactor chambers, and a 3D-printed frame for holding the membrane, is presented in Figure 1a. Each of the two bioreactor chambers was built up by two parts, a head and a body part. The head parts were the same for both chambers whereas the body parts were distinguishable by tongue and groove, which together with the tightly fitting clamps effectively sealed the reactors. A scheme of the three-step fabrication process of a head part is presented in Figure 1d. The parts printed by the SLA 3D printer had a smooth surface quality and after short post-processing times could be used for creating the negative molds. The molding silicone that was applied to generate the negative molds perfectly reproduced original geometries and features while curing at room temperature in less than 30 min. Thereby, enough negative molds could be fabricated by one 3D-printed part to facilitate a parallel production of several reactors. Another beneficial property of the molding silicone was its high flexibility after curing. This enabled the molding of undercut features, such as the tongue and groove, which granted the proper sealing of the reactors. A total of 2 min of oxygen plasma treatment was then sufficient to passivate the molds, and effectively prevented PDMS that was pumped into the molds from sticking. The head part was then bonded to one of the body parts by the stick-and-stamp method. After the assembly of these major parts, the bioreactor could be equipped with suitable Luer Lock tubing connectors and was closed by four specially designed 3D-printed clamps (Figure 1b). After its connection to the tubing system, the reactors were ready to use for dynamic in vitro culture (Figure 1c).

Once the details of the fabrication process were outlined, it was possible to achieve a time-efficient production of different parts. Here, crucial parameters were the duration of oxygen plasma treatment, the duration and curing temperature of PDMS, as well as the bonding procedure of different PDMS parts by the stick-and-stamp method.

The bioreactor holders (Figure 1e–g) were prepared by 3D printing and provided a secure upright positioning of the bioreactors. The upright positioning of the reactors proved to be advantageous to prevent air bubbles from being stuck in the bioreactor. The holders were mounted on silicone-casted platforms that prevented them from tilting or falling and, due to a soft sticking effect on the incubator floor, gave the reactor holders a firm stand.

Furthermore, the bioreactor design, using a printed rigid membrane holder that fitted tightly into the PDMS chamber of the reactors, on one hand kept the membrane mechanically fixed during fluid flow, and on the other hand separated the liquids of the two compartments. This effectively avoided cross-flow between the two compartments and thus electrical shortcuts that would otherwise distort the results of the EIS measurements.

By computational fluidic simulation, we confirmed that the chip bioreactor can generate stable laminar shear stress within the range of 1.76 × 10^−3^ to approximately 8.34 × 10^−2^ dyne/cm^2^ applied in fluid condition (Figure 2b).

### 3.2. Tube Electrode Development

In general, there are two options to place electrodes for EIS measurement into dynamic flow bioreactors. Either they are directly built into the bioreactor or they are separately constructed and then attached to the reactor periphery. In order to keep the fabrication process of the bioreactors as simple as possible, electrodes were developed that could be implemented in the tubing system outside of the bioreactor chamber harboring the tissues (Figure 3b). CAD drawings present the developed tube electrode in explosive view and in its assembled form in Figure 3a. After the assembly of the six individual components, which were all made from autoclavable materials, a PDMS shell was casted around this inner electrode part for additional sealing against leakage and electrical isolation (Figure 3a). Nano-rough TiN was used as an advanced electrode material due to its favorable material properties, such as high mechanical and chemical resistance, as well as low self-impedance [38,39]. Several steps of the fabrication process of the TiN-coated tube electrodes are presented in Figure 3c–e. The openings of the stainless steel tubes were parallel to the titanium target, and the coating process of the inner tube walls was performed via a so-called glancing angle deposition. PVD coatings deposited under glancing angle conditions tend to create a comparably rough surface structure, which could also be seen in the SEM image presented in Figure 3f.

### 3.3. Electrical Characterization of TiN-Coated Tube Electrodes

**Figure 5:**
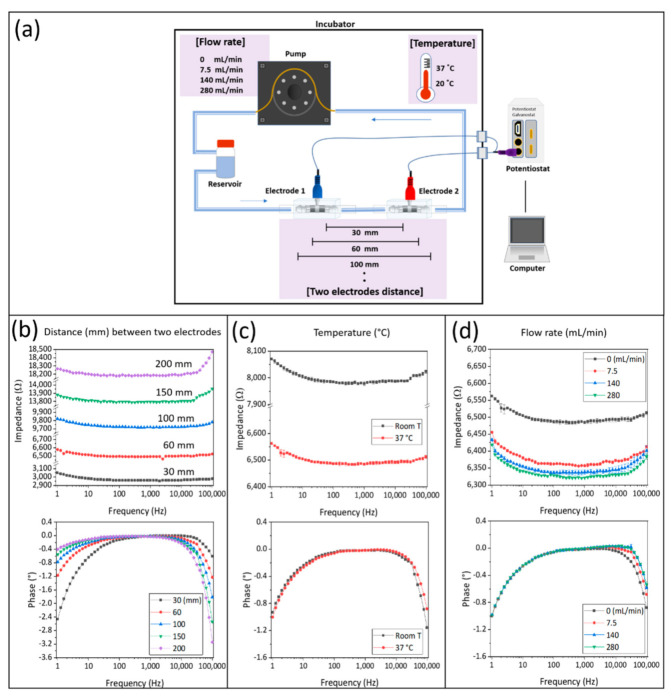
Characterization of electrical properties of the TiN tube electrodes. (**a**) A graphical image of electrochemical cell set up and test conditions for TiN tube electrode characterization. Bode blots of the recorded impedance spectra over the frequency, depending on (**b**) the distance between two electrodes at 30, 60, 100, 150, and 200 mm; (**c**) the temperature at RT (approximately 20 °C) and 37 °C, as well as (**d**) different flow rates of 0, 7.5, 140, and 280 mL/min.

In addition to the investigation of TiN coating surface structure, the electrical properties of the electrodes were characterized by EIS. The electrochemical cell used for this characterization comprised of two electrodes, connected by silicone tubing of varying lengths and with PBS^-^ as the measurement electrolyte. An equivalent circuit with a serial connection of a constant phase element (*CPE*) and a medium resistor (*R_m_*) could represent the test setup (Figure 5a). Three different parameters, the distance between two electrodes, temperature, and flow rate, were analyzed in separate experiments to validate the electrodes in the setup. The results of these parameter tests are represented as bode plots (Figure 5b–d). Here, in each recorded spectrum the impedance value at the frequency with the smallest phase shift (closest to phase angle 0°) is now referred to as impedance baseline.

As shown in Figure 5b, the impedance baseline increased with rising distance between the two electrodes from approximately 3000 Ω at 30 mm to approximately 18,200 Ω at 200 mm distance, respectively. In the recorded impedance spectra, increased amplitude and phase shifts at the lower and higher end of the spectra were observed. The first increase in amplitude and phase shift in the low frequency range from approximately 30 Hz to 1 Hz could be attributed to the increasing influence of the electrode material, which could be kept comparably low due to the nano-rough TiN coating. However, the influence of the electrode material on the measurement decreases with increasing frequency due to its higher capacitive behavior. At medium range frequencies, the resistance of the electrolyte determines the amplitude. Interestingly, a second increase in amplitude and phase shift could be observed with rising frequency, especially at measurement frequencies higher than 10 kHz. This rise in amplitude and phase shift was more pronounced and started at lower frequencies with the increasing distance of the electrodes. This can be explained by electrical resistivity. The electrical resistivity increased by increasing the tube length filled with electrolyte. As a result, the longer distance of the two electrodes showed higher amplitudes and more phase changes. Impedance was also affected by temperature and flow rate. The impedance baseline decreased with increased temperature, and the impedance difference was about 1500 Ω between room temperature and 37 °C, but phase changes were similar.

### 3.4. Impedance Spectroscopic Results

**Figure 6:**
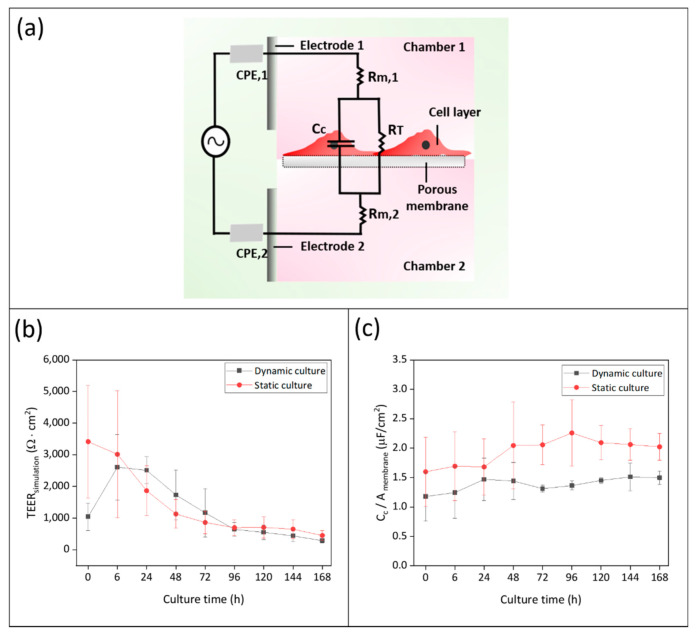
EIS measurement for monitoring of hiPSC-derived BCECs culture over 168 h in dynamic and static conditions (biological replicates N = 7 (0 h–48 h) and N = 3 (72 h–168 h)). (**a**) Equivalent circuit design based on the bioreactor culture system: *CPE* constant phase element of electrode, *Cc*: capacitance of cell membrane, *R_T_*: Resistance of tight junction barrier (*TEER_simulation_*), *R_m_*: resistance of culture medium; (**b**) results of fitted *TEER_simulation_*; and (**c**) cell membrane capacitance obtained by fitting and simulation of impedance data. Graphs show a comparison between dynamic and static culture over the period of culture.

After 10 days of differentiation, the membrane chips with hiPSC-derived BCECs were transferred from static culture conditions to the bioreactors to initiate dynamic culture (day 0 of dynamic culture) (Figure 4a). As soon as the whole bioreactor system was filled with medium, EIS measurement was carried out using the TiN tube electrodes connected to the potentiostat (Figure 4b,c). After the first measurement, the dynamic culture was monitored every 24 h. Simultaneously, we carried out EIS measurements of static Transwell-based models using a measuring plate with TiN electrodes to compare cellular barrier properties in different culture conditions. The TEER values of the tight junction barriers and the capacitance of the cell membranes were quantified by fitting equivalent circuits. Figure 6a shows an equivalent circuit, which is corresponding to our cell culture system, comprising resistors representing the cell culture medium (*R_m_*), constant phase elements (*CPE*) representing the electrodes, and a parallel connection of a resistor and a capacitor representing the tight junction barriers and lipid bilayer of the cells, respectively (Supplementary Figure S1). As a result, the TEER values (represented by the resistor *R_T_*) were displayed over culture time in dynamic and static culture (Figure 6b, Supplementary Figure S2).

At the 0 h measurement there were significant differences between the dynamic and static culture system, with TEER values of 1042 ± 433 Ω·cm^2^ and 3411 ± 1779 Ω·cm^2^, respectively. We confirmed that the adaption of the cells in the BR system to dynamic culture conditions caused a significant reduction of the tight junction barrier of hiPSC-derived BCECs (Supplementary Figure S2 and Table S1). Post-stabilization, the TEER values at 6 h reached 2609 ± 1029 Ω·cm^2^ under dynamic culture conditions. At 24 h of static culture, the TEER values were 1865 ± 786 Ω·cm^2^, while the dynamic culture achieved higher TEER values of 2513 ± 424 Ω·cm^2^. Afterwards the TEER values steadily dropped to 449 ± 149 Ω·cm^2^ and 285 ± 76 Ω·cm^2^ during static and dynamic culture, respectively, until a total culture time of 168 h was reached. Equivalent circuit fitting and simulation was not only applied to quantify TEER values but also the capacitance of the cell layer. Since cell culture areas between dynamic (0.95 cm^2^) and static (0.33 cm^2^) are different, capacitance values derived from electrical circuit equivalents were divided by surface area for each condition, this was done so since capacitance is proportional to the geometrical surface area. (Figure 6c). In dynamic culture, the capacitance increased slightly from 1.18 ± 0.41 µF/cm^2^ to 1.47 ± 0.36 µF/cm^2^ for 24 h, followed by stabilization during culture time, reaching a final value of 1.49 µF/cm^2^ at 168 h. In static culture, the capacitance at 0 h was 1.59 ± 0.58 µF/cm^2^ and increased to 2.05 ± 0.33 µF/cm^2^ at 72 h. It reached its highest value of 2.25 ± 0.56 µF/cm^2^ at 96 h with a minor decrease to 2.02 ± 0.22 µF/cm^2^ at 168 h. Overall, the capacitance of the cell layer during static culture was higher than during dynamic culture.

### 3.5. Expression of Characteristic Proteins

**Figure 7:**
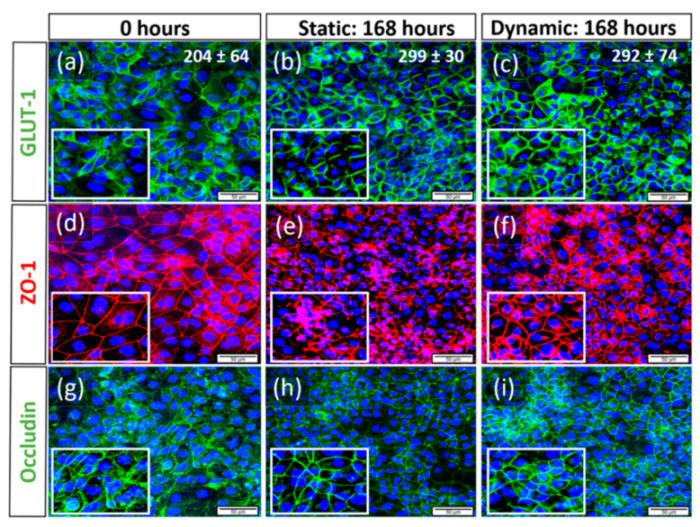
Changes of cell morphology and protein expression of hiPSC-derived BCECs compared between dynamic and static culture conditions, representative images of N = 3 biological replicates, scale bar = 50 µM, magnification = 40×. Via immunofluorescence, the expression of the Glucose transporter-1 (GLUT-1, (**a**–**c**)), as well as the TJ proteins Zonula Occludens-1 (ZO-1, (**d**–**f**)) and Occludin (**g**–**i**), was observed. In both 168 h static and dynamic conditions, BCECs showed more nuclear compaction and increased expression of relevant proteins such as GLUT-1 (**b**,**c**), ZO-1 (**e,f**), and Occludin (**h**,**i**), respectively, when compared to 0 h static conditions (**a,d,g**). Zoomed in frames show more cytoplasmic localization of GLUT-1 (**a**) at day 0 when compared to 168 h dynamic conditions (**c**). In all markers, we observed more pronounced protein localization at the cellular borders post-168 h of static (zoomed in frame (**b**,**e**,**h**)) and dynamic conditions (**c**,**f**,**i**). However, BCECs cultivated under 168 h of flow showed more continuous and smooth membranous staining patterns, while BCECs of long-term static culture represented discontinuous staining patterns. The cell numbers counted for each culture condition are inserted in the upper right corner of the respective images (**a**–**c**).

To identify the influence of shear stress induced by dynamic flow culture conditions on hiPSC-derived BCECs compared to static culture, and to analyse resulting changes on protein expression, we investigated the expression of the TJ proteins Zonula Occludens-1 (ZO-1, Figure 7d–f) and, Occludin (Figure 7g–i), as well as Glucose transporter-1 (GLUT-1, Figure 7a–c), via immunofluorescence. At day 0, before the cells were shifted into long-term conditions, we observed that hiPSC-derived BCECs expressed the analyzed markers homogeniously (Figure 7a,d,g). This was already reported previously [5,26]. After an additional 168 h in dynamic and static culture, we observed that BCECs were more compact and densely packed compared to the starting time point, and the sizes of the cells were much smaller. For each culture condition, nine images captured with 40× magnification of DAPI-stained cells were randomly selected for image automated analyses. Cell counting revealed that in comparison to day 0, the number of cells increased from 204 ± 64 to 299 ± 30 under static conditions and to 292 ± 74 under dynamic conditions, respectively (Figure 7a–c).

Additionally the staining patterns showed that tight junctions, (TJ) such as ZO-1 and Occludin, are more pronounced at the cellular borders in 168 h dynamic conditions with smoother and thinner staining patterns (zoomed in areas of Figure 7f,i). The same was true for GLUT-1, especially in 168 h dynamic conditions (zoomed in area, Figure 7c) when compared to more cytoplasmic localization at time point 0 (Figure 7a). Although the static 168 h condition showed a similar staining pattern,6 as seen with dynamic, it was not as distinct and many areas showed low/no expression of markers with no continuous membranous expression (Figure 7b,e,h).

## 4. Discussion

In this study, we fabricated a cost-efficient, highly reproducible, and easy handling bioreactor that allowed for a non-invasive monitoring of cellular barrier integrity during dynamic culture conditions. We could successfully culture hiPSC-derived BCECs for 168 h and non-invasively monitor barrier integrity via EIS.

Comparison of static and dynamic culture of barrier tissues with the addition of shear stress is technically challenging. Reported fluidic systems are complicated to setup and require specialized equipment [27,40,41]. In addition to being expensive compared to the standard static Transwell-based models, the throughput of these systems remains typically very low [42]. Therefore, cost-efficient, reproducible, controllable fluidic parameters, and non-invasive quality control measurements are highly required for advancing in vitro barrier tissue models [43,44].

Here, we demonstrated that the synergic usage of CAD, 3D printing, and silicone molding provided a cheap and easily fabricated bioreactor system. Our tested system allowed rapid production and personalized modifications to meet both engineering demands and biological application. The combination of the applied methods enabled short iteration times between prototype testing, and the production of final bioreactor versions at short notice. The development process was further accelerated by the parallel testing of different component designs, for example regarding the size and shape of tongue and groove to effectively close the reactor and thus prevent the leakage of media.

Due to the parallelization capability of the 3D printing processes, the possibility to prepare a suitable amount of silicone molds, and its subsequent multiple usage, the process made it also easy to fabricate batches of bioreactors together with the necessary 3D-printed accessories.

PDMS, as the material of choice for the bioreactor, offered several key advantages. From an engineer’s view, it is easy to process, and although ten times as expensive as the molding silicone, the material costs for each reactor remained low compared to milled parts or bioreactors made of glass. Due to its hardness when cured, it offered enough mechanical stability for the reactors to maintain their shape when pressed by the tightly fitting 3D-printed clamps, but supported sufficient flexibility that embodied undercut features, such as the sealing rings, which were able to interlock when the two chambers were pressed together.

From a user’s view, this results in the easy assembly and handling of the reactors under sterile conditions without the requirement of additional tools or screws. Importantly PDMS has been shown to be a biocompatible material, which is applicable for cell culture. PDMS can be sterilized by autoclaving and the reactors could be easily cleaned after their application in cell culture experiments. Therefore, the reactors were also appropriate for multiple usage. Furthermore, the usage of a separate adjustable frame offers the possibility to implement a large variety of possible scaffolds used in tissue culture. This includes for example electro-spun biomaterials, biological matrices, or gel-based matrices.

In addition to the bioreactor, we developed an online-monitoring system to control the barrier integrity of in vitro models by the integration of electrodes for EIS measurement. Several studies have introduced electrodes placed inside of the bioreactors to monitor tissues under dynamic culture conditions. Wang et al. embedded an Ag/AgCl electrode in the channel of the microfluidic bioreactor developed for coculture of mouse brain microvascular endothelial cells (b.End3), pericytes, and with/without C8-D1A astrocytes [40]. Griep et al. have reported that they fixed the Platinum (Pt) electrode using an optical adhesive on the PDMS chamber slides of a microfluidic device for the TEER measurement of immortalized human brain endothelial cell line hCMEC/D3 in dynamic culture conditions. In addition, Cacopardo et al. have shown that the electrode material, a silver-enriched epoxy paste, was dispensed on glass slides, which are a part of the bioreactor, using a computerized numerical control (CNC) machine for the cellular monitoring of Caco-2 monolayer culture [45]. In our study, to simplify the implementation of the electrodes into a bioreactor system, we aimed to produce electrodes as an independent unit. Specifically designed and 3D-printed connectors, together with the casted PDMS shells, not only effectively prevented leakage but also enabled easy and tight connections of the TiN-coated stainless steel electrodes to the silicone tubing of the fluidic circuit. A further benefit of the TiN-coated electrodes as independent units is their simple applicability to pre-existing fluid flow bioreactors that was also ensured by the design of the electrode setup.

However, the main advantage of TiN-coated electrodes was the low impedance of the electrodes themselves, which minimized their impact in EIS measurements, as demonstrated previously [31]. The influence of the measurement electrodes on the impedance signal is only noticeable in very low frequency ranges below 10 Hz. The electrical properties were determined and modeled in the electrical equivalent circuit. Furthermore, the electrical characterization of the developed electrode setup was performed by variation of crucial parameters, namely (I) distance between the electrodes in the system, (II) temperature, and (III) flow speed. It was shown that the TiN electrodes provided stable and sensitive impedance detection within the most relevant frequency range (1 Hz to 100 kHz).

Since we aimed to produce independent electrode units, there was a comparably large distance of approximately 20 cm between the two measurement electrodes, evoked by the size of the bioreactor. Increasing distance between the electrodes resulted in an increase of the impedance values, and the larger electrode distance resulted in a shift to a higher baseline. This was modeled by higher values for *R_m_* in the electrical equivalent circuit. We also confirmed that the variation of temperature and flow rate caused a change of the baseline value of *R_m_*. This means that culture conditions, such as temperature and flow rate, should be maintained as stable as possible during EIS measurement. However, all of these above-mentioned factors are controllable during the cell culture experiments, so the value for *R_m_* can be precisely determined from the impedance data.

Since the values of the *CPE* and *R_m_* in the electrical equivalent circuit were known or could be directly acquired from the impedance data, the influence of the monitored cell culture model on the impedance spectra was clearly determinable at any time point of in vitro culture.

To test the reliability and reproducibility of the complete monitoring bioreactor system, hiPSC-derived BCECs were cultured under controlled flow and culture conditions (37 °C and 5% CO_2_) for up to 168 h. Computational fluidic simulation revealed that a steady laminar shear stress can be controlled in the range of 1.76 × 10^−3^ to approximately 8.34 × 10^−2^ dyne/cm^2^. For this first test of the reactor system, the lowest value of this range was applied to achieve stable and reproducible conditions. Reports by Vatine et al. have shown that hiPSC-derived BCECs are already affected by low shear stress conditions of 0.01~2.4 dyne/cm^2^ induced by laminar medium flow. Their study indicates that laminar flow promotes the expression of pathways related to BCECs maturation [46]. Meanwhile, DeStefano et al. have shown that the introduction of shear stress in the range of 4~12 dyne/cm^2^ on hiPSC-derived BCECs resulted in no change in the TJ expression at gene and protein level [47]. In our study, a shear stress of 1.76 × 10^−3^ dyne/cm^2^ influenced the morphology of hiPSC-derived BCECs within 168 h of in vitro culture. BCECs cultivated under dynamic conditions displayed smaller and condensed nuclei, and characteristic expression of TJ proteins (ZO-1 and Occludin), as well as GLUT-1, at the cellular borders.

During EIS, the magnitude of voltage was kept low (50 mV) to prevent the involuntary physiological changes of cells and cell death [48] that may occur because of an electrical current supply. The recorded EIS measurements were quantified by fitting the equivalent circuits to determine the elements in the circuit in the most accurate way. Concerning in vitro BBB modelling, it is crucial that barrier integrity is monitored non-invasively and quality controlled, especially if such a system should be used in pharmaceutical testing. If there is a break in cellular monolayers due to cellular damages, this would lead to faulty assumptions of drug permeability values. However, layer peeling can be instantly recognized by EIS online monitoring as a drop of the TEER value to 0 Ω·cm^2^ or to the system’s baseline value in the recorded impedance spectra, respectively.

In contrast to the expression of TJ proteins, the TEER values of hiPSC-derived BCECs in dynamic culture were quite comparable to the static culture, with final values of 285 ± 76 Ω·cm^2^ and 449 ± 149 Ω·cm^2^ at 168 h of in vitro culture. This is in line with reports from Hollmann et al., who demonstrated that BCECs show a decrease in TEER with increased static culture durations [49]. We observed no statistical significances in TEER values comparing both static and dynamic culture. However, BCECs have to adapt to new culture conditions during dynamic flow, thereby showing a TEER of 1042 ± 433 Ω·cm^2^ at time point 0 h, followed by a revival of barrier integrity at 6 h with a TEER of 2609 ± 1029 Ω·cm^2^. Interestingly, a high variability of TEER at 0 h is noted in different biological replicates after 10 days of differentiation (raw data included in Supplementary Table S1), which could be due to variations in differentiation efficacy [50].

Although the TEER values obtained in our study are comparatively low at time point 168 h, with values of 285 ± 76 Ω·cm^2^ in dynamic and 449 ± 149 Ω·cm^2^ in static cultured BBB models, protein expression via immunofluorescence indicates that the cells have more membranous and continuous tight junction expressions, hypothesizing BCEC maturation. However, further molecular-based studies are required to identify the reasons for these observations.

Previous studies have shown that not only TEER but also cell membrane capacitance can be used as useful indicators for the non-invasive monitoring of cell properties, such as differentiation [51,52,53], morphological changes [54], motility [55], and mortality [56]. In this study, the cell membrane capacitance of BCECs could be acquired by fitting an equivalent circuit using obtained impedance data. The capacitance slightly increased over culture time from 1.18 ± 0.41 µF/cm^2^ at 0 h to 1.49 µF/cm^2^ at 168 h in dynamic and 1.59 ± 0.58 µF/cm^2^ at 0 h to 2.02 ± 0.22 µF/cm^2^ at 168 h in static culture.

The increase in capacitance during culture time in both systems could be associated with the increasing number of cells compared to day 0 that was revealed by nuclei counting. In both culture systems, this increase in cell number was accompanied by a change in BCEC morphology that could be visualized in cytoplasmic compaction and the increased membranous expression patterns of TJs. This indicated that the spread of BCECs was reduced in comparison to day 0, reflecting a transition from larger wide-spread cells to smaller and more compact cells. Since capacitance is strongly connected to the cell membrane surface area [57,58], the lower values in the dynamic system could be attributed to an overall smaller membrane surface area of the BCECs compared to the static system. However, this could not be directly linked to dynamic effects such as shear stress, since the dynamic capacitance was already lower from the start of the culture at 0 h.

The growing cell numbers could also have an impact on the decreasing TEER values in both models. The increasing number of cells leads to an increasing number of cell borders and therefore pathways for ions to pass the cellular barrier [59]. However, the scale of the decrease regarding the TEER values makes it more likely that the hypothesized maturation of the BCECs continuously lowered the overall barrier integrity regarding their permeability for ions. Notably, decreasing TEER values are not necessarily connected to a higher permeability regarding other non-electrolyte paracellular tracers, such as fluorescein isothiocyanate (FITC)-labeled dextrans for example, since the two experimental approaches depend on different transport mechanisms through the barrier [60]. Further studies addressing the connection between the maturation and morphological changes of BCECs, as well as the electrical parameters and the permeability of tracer molecules, have to be conducted to address this question.

Overall, we were able to show that our bioreactor system is appropriate for the non-invasive online monitoring of the cellular barrier models. This dynamic culture system can be applied for long-term culture of shear stress-sensitive in vitro tissue models.

## 5. Conclusions

In conclusion, our novel bioreactor system with externally attachable electrodes provides a non-invasive and automated platform to monitor the barrier integrity of tissue models online. By the application of dynamic flow culture conditions, a more physiological environment is simulated in order to mechanically stimulate shear stress-sensitive cells. By aid of the presented system, we are providing valuable information in the identification and monitoring of cellular barrier of hiPSC-derived BCECs as one example of flow-sensitive cellular systems. With a two-separated fluid regime of the bioreactor system, a complex co-culture model could be carried out to improve the model one step further toward the human in vivo-like environment [26]. Furthermore, our model enables the possibility to perform transport studies under dynamic flow culture conditions in the future, which are important, for example, in flow-dependent infection and drug transport studies.

## Data Availability

The data presented in this study are available on request from the corresponding author.

## Supplementary Materials

The following are available online at https://www.mdpi.com/article/10.3390/bioengineering9010039/s1, Figure S1: Equivalent circuit element represents the chip bioreactor system; Figure S2: EIS data in bode plot for 168 h of dynamic and static culture; Table S1: overview tables of TEER and cell membrane capacitance values.
