# Supplementary material for: Online Measurement System for Dynamic Flow Bioreactors to Study Barrier Integrity of hiPSC-Based Blood–Brain Barrier In Vitro Models
Source: Bioengineering (Basel). 2022 Jan 16;9(1):39. doi: 10.3390/bioengineering9010039 (PMC8773345; doi:10.3390/bioengineering9010039)
Supplement: Supplementary file 1 [file bioengineering-09-00039-s001.zip › bioengineering-1505095-supplementary.pdf]

bioengineering MDPI

Supplementary materials

Online Measurement System for Dynamic Flow Bioreactors to Study Barrier Integrity of hiPSC-Based Blood–Brain Barrier In Vitro Models

Jihyoung Choi [1,#,] *, Sanjana Mathew [1,#], Sabrina Oerter [2], Antje Appelt-Menzel [1,2*], Jan Hansmann[1,3] and Tobias Schmitz [1]

[1] Department of Tissue Engineering and Regenerative Medicine, University Hospital Würzburg, Röntgenring 11, 97070 Würzburg, Germany; sanjana.mathew@uni-wuerzburg.de (S.M.); Jan.Hansmann@uni-wuerzburg.de (J.H.); tobias.schmitz@uni-wuerzburg.de (T.S.)
[2] Translational Center for Regenerative Therapies, Fraunhofer Institute for Silicate Research , Röntgenring 11, 97070 Würzburg, Germany; sabrina.oerter@isc.fraunhofer.de
[3] Faculty of Electronics, University of Applied Science Würzburg-Schweinfurt, Ignaz-Schön-Straße 11, 97421 Schweinfurt, Germany
* Correspondence: jihyoung.choi@uni-wuerzburg.de (J.C.); antje.appelt-menzel@uni-wuerzburg.de (A.A.-M.)
† These authors contributed equally to this work

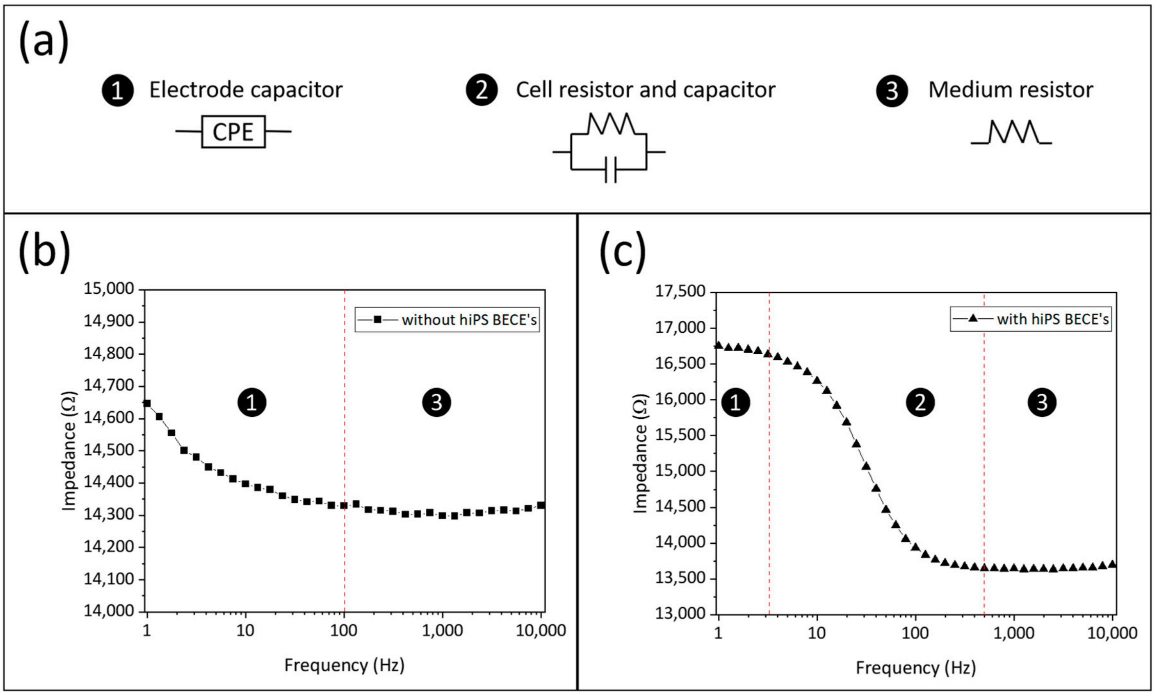

Figure S1. Equivalent circuit elements that represent the chip bioreactor system. (**a**) Three main elements of equivalent circuits representing the bioreactor cell culture system. Comparison between EIS measurement (**b**) without hiPSC-derived BCECs and (**c**) with hiPSC-derived BCECs culture on porous membrane in chip bioreactor system. The simulated electronic parameters for the *CPE* in the dynamic system were: Exponent of *CPE* N = 0.65 ± 0.09 and coefficient of *CPE* $Y_0 = 88.4 \pm 44.2 \cdot 10^{-5}\ \Omega^{-1} \cdot s^N \cdot cm^2$.

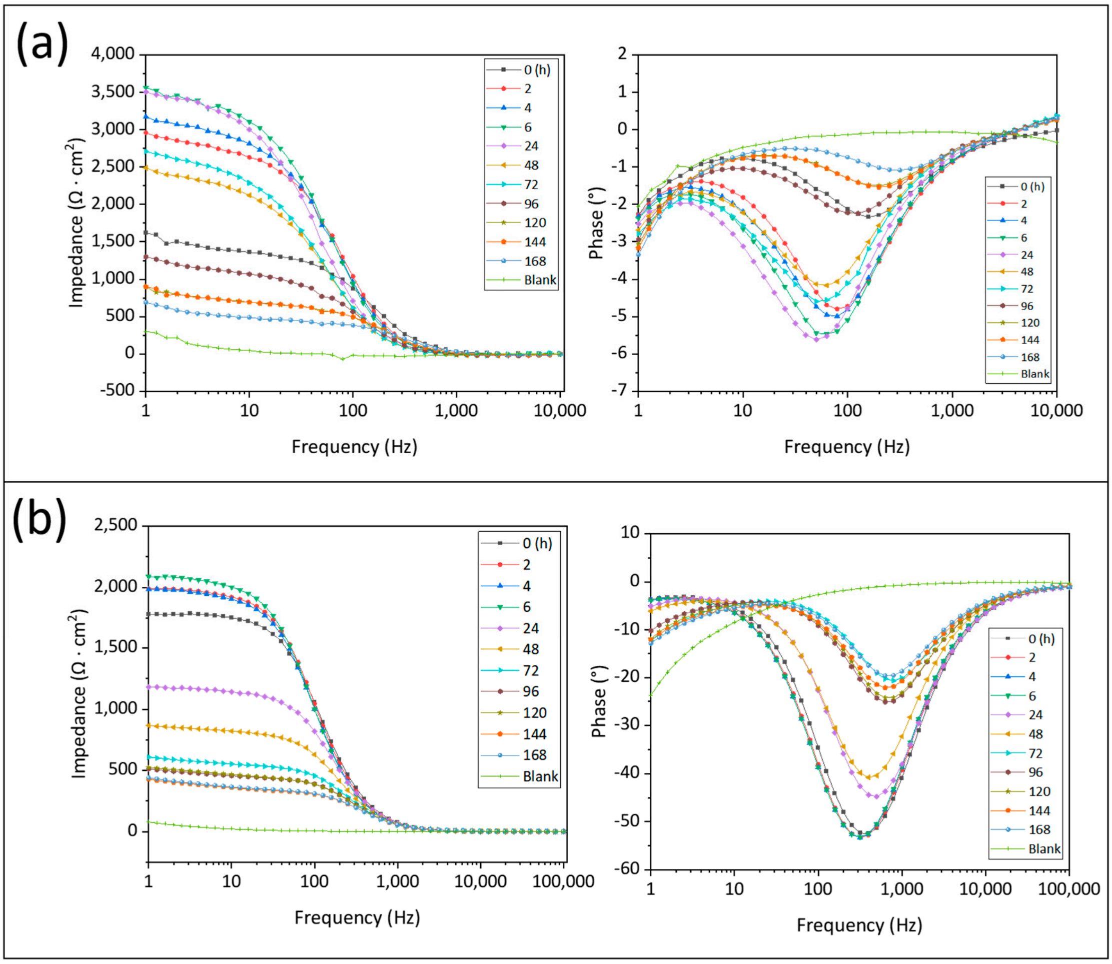

Figure S2. Impedance of frequency respond was represented in Bode plot. We subtracted baseline and then normalized impedance value by porous membrane area (dynamic culture ~ 0.95 cm^2, static culture ~ 0.33 cm^2) for better comparison. EIS measurement carried out at time points (0, 2, 4, 6, 24, 48, 72, 96, 120, 144, and 168 h) for 168 h BCECs culture (**a**) in dynamic culture condition and (**b**) static culture condition. The simulated electronic parameters for the *CPE* in the static system were: Exponent of *CPE* N = 0.39 ± 0.06 and coefficient of *CPE* $Y_0 = 85.3 \pm 39.1 \cdot 10^{-5}\ \Omega^{-1} \cdot s^N \cdot cm^2$.

Table S1. Overview of transendothelial electrical resistances (*TEER*) and cell membrane capacitance (C_c) of BCECs in static culture in transwells and dynamic culture in bioreactor system. Cellular barrier impedance was monitored by EIS. TEER and C_c were quantified by fitting equivalent circuits. 168 h dynamic and static culture (N = 3). 48 h dynamic and static culture (N = 4), resulting in (N = 7) for all measurement time points until 48 h.

	$TEER_{simulation}$ (Ω·cm²)				$Cc/A_{membrane}$ (μF/cm²)			
	Dynamic culture		Static culture		Dynamic culture		Static culture	
**Culture time (h)**	Mean	SD	Mean	SD	Mean	SD	Mean	SD
0	1,042	433	3,411	1,779	1.18	0.41	1.59	0.58
2	2,108	762	3,143	1,984	1.06	0.35	1.63	0.58
4	2,431	1,037	3,219	1,992	1.11	0.37	1.68	0.59
6	2,609	1,029	3,010	2,008	1.16	0.37	1.69	0.58
24	2,513	424	1,865	786	1.47	0.36	1.68	0.47
48	1,733	785	1,129	451	1.44	0.31	2.04	0.73
72	1,165	762	860	358	1.31	0.05	2.05	0.33
96	641	219	697	244	1.36	0.07	2.25	0.56
120	550	232	706	336	1.45	0.04	2.09	0.29
144	437	176	648	298	1.51	0.23	2.06	0.26
168	285	76	449	149	1.49	0.11	2.02	0.22
